# Hybrid Single-Port Cholecystectomy of a Pediatric Gallbladder Volvulus

**DOI:** 10.7759/cureus.23801

**Published:** 2022-04-04

**Authors:** Wesley J Lemons, Rachel Desimone, Federico G Seifarth

**Affiliations:** 1 Pediatric Surgery, West Virginia University School of Medicine, Morgantown, USA; 2 Pediatric Surgery, Logan Healthcare, Kalispell, USA

**Keywords:** pediatric laparoscopic surgery, single-port laparoscopic surgery, lap chole, gallbladder volvulus, gallbladder removal

## Abstract

Gallbladder volvulus is a rare gallbladder pathology that can present in adults but is exceedingly rare in children. The diagnosis itself can be very challenging due to its presentation with signs and symptoms of acute cholecystitis without specific imaging findings. The correct identification and prompt intervention with a cholecystectomy are crucial to improve patient outcomes. In this report, we discuss a pediatric patient who presented with gallbladder volvulus and subsequently underwent novel treatment with a single-port laparoscopic cholecystectomy.

The patient is a 12-year-old male of Haitian descent who presented to an outside facility following the onset of persistent, right upper quadrant abdominal pain and recurrent nonbilious emesis. The diagnostic workup included serial abdominal exams, laboratory work, right upper quadrant ultrasounds, and a hepatobiliary iminodiacetic acid (HIDA) scan. The patient then underwent single-port laparoscopic removal of the torsed gallbladder with complete resolution of his symptoms. In this case report, the management and clinical presentation of gallbladder volvulus are discussed in more detail as well as the feasibility of single-port laparoscopic cholecystectomy in the setting of pediatric gallbladder volvulus.

## Introduction

Gallbladder torsion is exceedingly rare in the pediatric population as the median age at presentation is 77 years [1]. It is defined by the rotation of the gallbladder along the axis of the cystic duct and the vascular pedicle. Pre-operative diagnosis is very challenging as patients present with clinical symptoms similar to acute cholecystitis, often with nonspecific imaging and laboratory findings. The mortality rate of gallbladder torsion is estimated at 5%, even with early surgical intervention [1,2]. Single-port laparoscopic surgery is a useful tool for the diagnosis and treatment of this surgical condition and has not yet been described in this clinical setting. This surgical technique benefits the patient by reducing incisional sites and providing excellent cosmesis compared to the standard four-port approach.

## Case presentation

A 12-year-old male of Haitian descent with no significant past medical history presented to urgent care with a two-day history of persistent, right upper quadrant abdominal pain and recurrent nonbilious emesis. The complaints were not associated with food intake. The initial workup at an outside facility consisted of an abdominal ultrasound that showed evidence of gallbladder dilation and gallbladder wall thickening of up to 6 mm in diameter. No stones, sludge, or common bile duct dilation were identified at this time. Laboratory evaluation was unremarkable with a normal white blood cell count, normal liver enzymes, and gamma-glutamyl transferase. The workup at this time was concluded to be negative, and the patient was sent home.

The patient re-presented within 24 hours with worsening right upper quadrant pain, persistent nonbilious emesis, and new onset of low-grade fevers. Abdominal ultrasound and lab work were repeated at this time, which demonstrated similar findings with the exception of an interval rise of C-reactive protein (CRP) to 6.4 mg/dL. He was admitted with presumed cholecystitis for observation and further workup. Ampicillin/sulbactam therapy was initiated, and he was kept NPO (nothing by mouth). Overnight, his nausea resolved, but his abdominal pain in the right upper quadrant persisted. Due to the presence of clinical findings with incongruent imaging, a hepatobiliary iminodiacetic acid (HIDA) scan with a cholecystokinin (CCK) injection was performed. The HIDA scan resulted in no visualization of the gallbladder, compatible with cystic duct obstruction. After the HIDA scan and a third focused ultrasound, he was taken to the operating room for exploratory laparoscopy with the working diagnosis of acalculous cholecystitis. 

**Figure 1:**
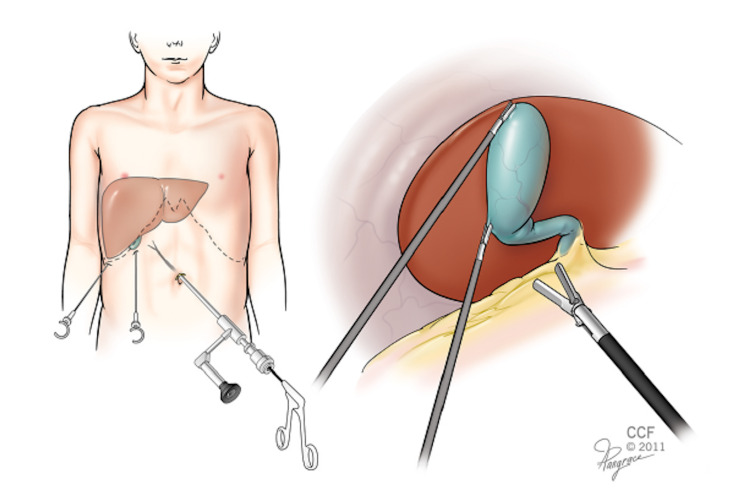
10-mm Hopkins telescope with inbuilt working channel Image source: The figure was taken from Ref. [3].

A 12-mm Step™ Bladeless Trocar (Covidien, Mansfield, MA) was placed into the peritoneal cavity through a single vertical, trans-umbilical incision. After establishing a pneumoperitoneum to 12 mmHg, the Storz 10-mm Hopkins telescope with an inbuilt working channel was introduced (Figure 1).

**Figure 2:**
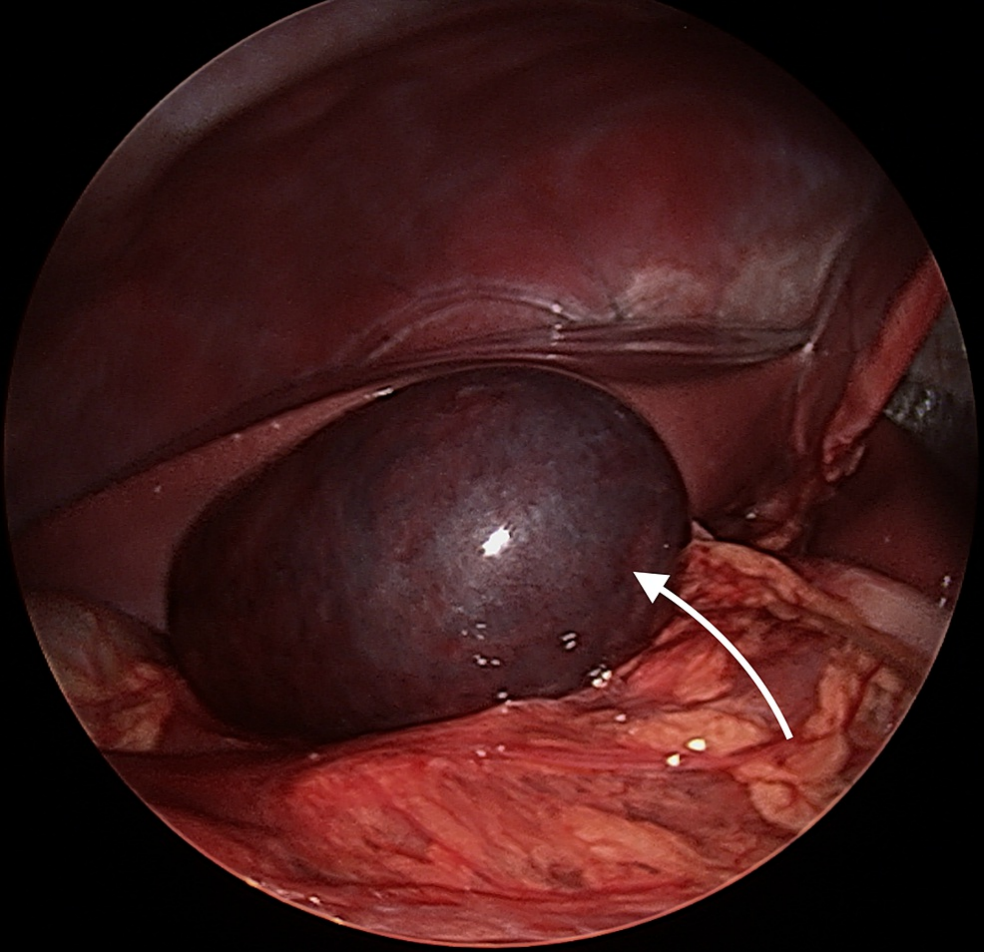
Laparoscopic view of the inflamed and gangrenous gallbladder

Operative findings were significant for a gangrenous and massively distended gallbladder (Figure 2). Two additional 2-mm stab incisions were made along the right costal margin. A laparoscopic suction needle was passed through one of the incisions, and approximately 30 ml of hemorrhagic-bilious fluid was evacuated from the gallbladder. A laparoscopic Weck Hem-o-Lok® clip (Weck® Closure Systems, Research Triangle Park, NC) was used to seal the puncture hole, and two blunt 2.3-mm clutch graspers were subsequently passed through the mini stab incisions to manipulate and expose the gallbladder.

**Figure 3:**
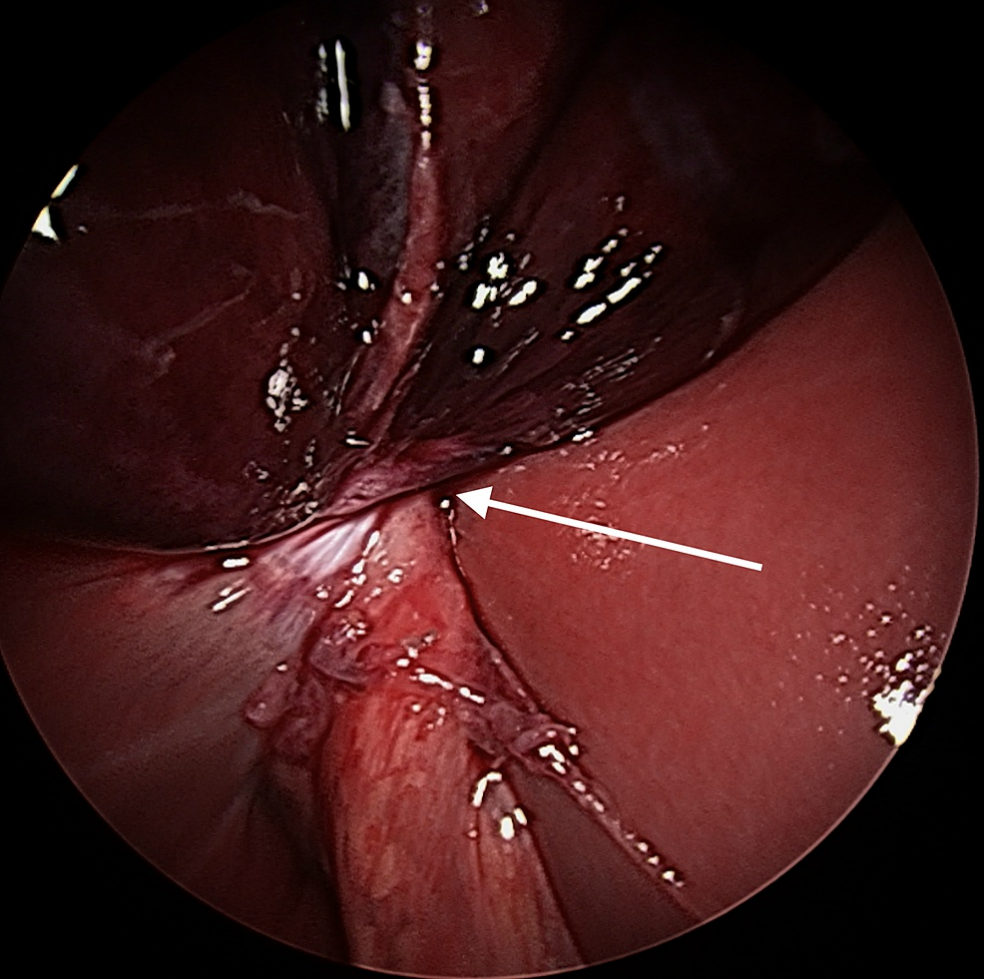
Laparoscopic view of the twisted gallbladder with minimal hepatic attachments

Upon evaluation, the gallbladder showed minimal hepatic attachment, and the infundibulum was found to be twisted (Figure 3). The gallbladder was de-rotated 270 degrees (counterclockwise), which resulted in partial resolution of the dark discoloration as visual evidence of reperfusion. The peritoneal lining around the infundibulum was incised with the hook electrocautery, and the cystic artery and duct were doubly clipped using the Hem-o-Lok clips. A 2 cm x 2 cm large hepatic gallbladder adhesion was divided with electrocautery, and the gallbladder was extracted via the umbilical incision. The gallbladder pathology report noted a hemorrhagic, partially necrotic, and thickened wall and no evidence of cholelithiasis.

The patient was discharged home on postoperative day one with standard oral pain management of acetaminophen, ibuprofen, and oxycodone. He was tolerating a low-fat diet with complete resolution of his right upper quadrant pain within days. He did not present for surgical follow-up.

## Discussion

Gallbladder volvulus is remarkably rare in children with less than 60 reported cases in the world literature [1-6]. The first pediatric case was described in 1925 by Daux, with subsequent reports stressing the diagnostic challenge and high mortality if not promptly treated [7,8]. The diagnosis of gallbladder volvulus has an estimated mortality rate of 5% even with early diagnosis and surgical intervention, which is notably higher than the estimated 3.6% mortality of acute cholecystitis [9]. The increased mortality of gallbladder volvulus is attributed to strangulation of the cystic artery, which progresses to gallbladder necrosis and perforation with a near 100% mortality rate [10]. Since its first description in 1898, around 500 cases of gallbladder torsion have been reported in the literature with a median age of 77 years [1,2,11]. Though well known in the adult population, the diagnosis of gallbladder volvulus is exceedingly rare in the pediatric population.

The etiology of gallbladder volvulus is still unclear. A redundant mesentery and lack of gallbladder adhesions to the liver, leading to a “floating gallbladder,” are predisposing factors [1,12]. It is speculated that the loss of visceral fat in the elderly helps explain the higher incidence in the older age group compared to the pediatric population [12]. In adults, gallbladder volvulus is more common among women, occurring at a female:male ratio of 4:1. In children, this condition was found to be more common in males than females with a 2.5:1 ratio [1,2].

As with many gallbladder pathologies, the key finding for gallbladder torsion or volvulus during the history and physical is abdominal pain. The symptoms can range from intermittent right upper quadrant discomfort with nausea and vomiting and mimicking biliary colic to the severe clinical picture of an acute abdomen. In light of this nonspecific presentation, diagnosis can be very challenging. Depending on the degree of torsion and length of symptoms, laboratory findings can be equivocal and radiographic signs subtle [13]. Abdominal ultrasound (US) is the accepted standard for gallbladder evaluation. US findings are generally nonspecific and consistent with gallbladder distension, wall thickening, and sometimes pericystic fluid [13]. If inconclusive, radiologic evaluation can be escalated to computed tomography (CT) or magnetic resonance imaging. The “cystic duct sign” is an infrequent finding caused by the nodular appearance of the swirled cystic duct, artery, and gallbladder neck. Additionally, abnormal anatomical position and lack of hepatic attachments can be noticed, which are referred to as “floating” gallbladder [11]. In this case, ultrasound was repeated due to its high sensitivity for gallbladder pathology and no radiation exposure, which are additional considerations in the pediatric population. On repeat exam, the patient presented with indications for laparoscopic exploration, which was performed using the single-port system.

About one-third of all patients presenting with gallbladder volvulus also present with concomitant cholelithiasis; therefore, gallstones are considered an incidental finding and not contributory to the etiology [1,2]. Our patient had three consecutive ultrasounds that revealed persistent gallbladder distension, gallbladder wall thickening, and pericystic fluid, leading to the working diagnosis of acalculous cholecystitis. Depending on the timing of the exam, a HIDA scan can show a “bullseye sign” created by accumulation and entrapment of radio-isotope within the gallbladder [12]. Our patient’s HIDA scan failed to visualize the gallbladder as a consequence of complete cystic duct obstruction from the volvulus at the time of the exam.

Laparoscopic cholecystectomy for gallbladder disease in children is considered the gold standard; its application for gallbladder volvulus has been described and deemed safe [4,6]. Some authors advocate for early laparoscopic exploration in the setting of clinical suspicion for gallbladder volvulus but lack radiographic evidence [6]. To our best knowledge, only rare reports of hybrid single-port cholecystectomy in a pediatric patient with gallbladder volvulus have been published. This single-port technique uses a straight 10-mm laparoscope with an off-site working channel [3]. This “all-in-one” instrument offers a minimally invasive surgical tool to diagnose and treat via a single umbilical port compared to a traditional laparoscopic approach, which requires a scope and additional separate ports for working instruments.

After establishing the diagnosis of gallbladder volvulus, meticulous dissection and detorsion of the gallbladder were performed with the help of two additional port-fewer mini-graspers. The single-port scope provides adequate visualization and excellent cosmetic results due to the barely visible incision. The minimally invasive nature of the single port makes surgical exploration more viable when faced with inconclusive imaging results. It is, however, limited by the 0-degree optic and the use of the mini-graspers with relatively small jaws. For advanced stages of torsion with gallbladder wall necrosis, conversion to the traditional four-port cholecystectomy with blunter 5-mm instruments is advised to prevent iatrogenic injury.

## Conclusions

Pediatric gallbladder volvulus is rare and presents with nonspecific, clinical, laboratory, and imaging findings. The pathology and diagnosis are not well known in the pediatric surgical field. Therefore, we advocate for early laparoscopic exploration in the case of nonspecific radiographic imaging with right upper quadrant pain. As stated, gallbladder volvulus has an estimated mortality rate of 5% and can progress rapidly to necrosis and perforation, which carries a near 100% mortality rate, making prompt diagnosis critical. The single-port 10-mm Hopkins telescope with an inbuilt working channel in combination with two portless mini-graspers is a reasonable alternative to traditional laparoscopy for diagnosis and cholecystectomy in the setting of pediatric gallbladder volvulus. Written patient consent for publication of this case report was obtained, and all patient information has been de-identified.
